# 

**Published:** 1882-03-20

**Authors:** 


### TABLE OF CONTENTS.

ORIGINAL AND SELECTED ARTICLES.

Viburnum Prunifolium Unreliable in Abortion, by A G Smythe, M. D., of Miss., 81. Movement as a Means of cure, 83. Treatment of Compound Fractures and Wounds of Joints, by F C G Griffin, M. D., of England, 87. Diphtheria and Bacteria, 90. Cases of Sephalic Version in the Postural Position, by John Havenstein, M. D., 92. Filaria Sanguinis Hominis, 94. Potassium Permanganate as an Antidote to the Venom of Serpents, by M. DeLacerda, 95.

ABSTRACTS AND GLEANINGS.

Asiatic Cholera and Yellow Fever, 97. The American Public Health Association, 99. Small-pox, 100. The Period of Gestation and the Placenta of the Elephant, 101. Curability and Treatment of Pulmonary Phthisis, 102. Small-pox and Vaccination, 103. The Use of Boracic Acid and Calendula in Ear Disease; The Treatment of Sea-Sickness, 104. Sulphide of Calcium in Strumous Ophthalmia, 105. Iodide of Potassium in Frontal Headache; Treatment of Wine-marks by Electrolysis, 106. Questions on Vaccination, 107. Treatment of Laryngeal Phthisis; Carcinoma of the Breast, 108. Acute Rheumatism; On the Removal of Warts; Vaccination in Scotland, 109. Oil of Wintergreen as an Effective Salicylate in Rheumatism; A New Cinchona Alkaloid; Hazeline as a Local Application, etc., 110.

SCIENTIFIC ITEMS.

The Agricultural Ant of Texas; Flowers in Sleeping-rooms, 111. Pulmonary Gymnastics; Milk to Extinguish Petroleum; Effects of the Electric Light on the Eyesight; A German Patent, 112.

PRACTICAL NOTES AND FORMULÆ.

Aloes for Piles; Styptic Colloid; Ergotine in Pharyngitis, 113. Mild Tonic Bitters; For Syphilis; Herpes Zoster; Kelly's Tonic, 114. Macdonald on Carbolic Acid in Whooping-cough; Hamilton's Tonic; Anti-Rheumatic Mixture; Effervescing Mixture, 115.

EDITORIALS AND MISCELLANEOUS.

Southern Medical College Commencement, 116. The American Medical Association; The Georgia Medical Association; Wm. R. Warner; Dr. C. L. Redwine; Patent and Trade Marks; Dr. Squibb, 119. Receipts; Special Notices, 120.

